# Ionic Liquid-Mediated Homogeneous Esterification of Cinnamic Anhydride to Xylans

**DOI:** 10.3390/ijms18122502

**Published:** 2017-11-23

**Authors:** Guihua Yang, Huifang Zhou, Jiachuan Chen, Gaojin Lyu, Yuanyuan Xia, Lucian A. Lucia

**Affiliations:** 1 Key Lab of Pulp & Paper Science and Technology of the Ministry of Education, Qilu University of Technology, Jinan 250353, China; ygh@qlu.edu.cn (G.Y.); huifangzhou2017@gmail.com (H.Z.); yuanyuanxia2017@gmail.com (Y.X.); lalucia@ncsu.edu (L.A.L.); 2 Departments of Chemistry, Wood & Paper Science (Forest Biomaterials), North Carolina State University, Raleigh, NC 27695-8005, USA

**Keywords:** homogeneous esterification, xylan, cinnamic anhydride, ionic liquids, functionalization, degrees of substitution

## Abstract

A new functional biopolymer was synthesized through an ionic liquid-mediated homogeneous grafting of cinnamic anhydride to xylans. The ionic liquid used was 1-allyl-3-methylimidazolium chloride (AMIMCl) ionic liquid. Xylans with degrees of substitution (DS) between 0.11 and 0.57 were accessible in a completely homogeneous system by changing catalysts (NaOH, KOH and LiOH), time, reaction temperature, and cinnamic anhydride/xylan molar ratio. The chemical structure and the thermal stability of the derivatives were characterized by Fourier transform infrared spectroscopy (FT-IR), ^13^C-NMR spectroscopy, and thermogravimetry. The thermal stability of the derivatives was reduced compared with the original xylan. Possible applications of the cinnamic anhydride-acylated xylan derivatives include wet-end papermaking, organic–inorganic composite films, and hydrogels.

## 1. Introduction

Currently, 270 million tons of petroleum is used globally per year for the manufacture of plastics [1]. The exploration of renewable alternatives to petroleum-based plastics is necessary given its anticipated depletion; hemicelluloses are an excellent alternative to provide a source of chemicals as already witnessed for furfural, xylitol, ethanol, and lactic acid [2,3,4]. Hemicelluloses, the non-cellulose polysaccharides in plant cell walls, represent 20–40% of total biomass reserves. Efficient use of these polysaccharides in preparing functional biopolymers and biomaterials will reduce dependence on fossil resources [5,6,7,8,9,10]. In fact, hemicelluloses have been used for the synthesis of many new bio-based materials, such as hydrogels, waterproofing materials, biomedical materials, film and coating materials, and wet-end papermaking additives [2,6,11,12].

To exploit new applications of hemicelluloses, many chemical modifications have been attempted [13]. For instance, lauroylated hemicelluloses [14,15,16], quaternized hemicelluloses [17,18], carboxymethylated hemicelluloses [14,15,16,19,20], acetylated hemicelluloses [17,18,21,22], and esterified hemicelluloses have been reported [23,24]. Ionic liquids (ILs), which have been widely promoted as “green solvents”, are attracting increasing attention as a reaction medium in carbohydrate polymer modification because of their chemical stability [25,26], eco-friendliness [27], high ionic conductivity properties, enhanced electrochemical and thermal stability, and high reaction rates [19,28,29,30,31]. However, information on the chemical modification of xylan, the major hemicellulose, in ILs is limited. Homogeneous solutions of xylan-rich hemicelluloses in ILs had been obtained and used for the acetylation of wheat straw hemicelluloses as early as 2007 [17]. Results showed that ILs could be used as satisfactory homogeneous media due to the lack of observed derivation during ILs dissolution.

Cinnamic anhydride (CA) modified polymers have been utilized. Anhydride CA can act as a super absorbent for immobilization of bioactive molecules and permselectivity of water [32], heavy metals [33,34], electrostatic adsorption self-assembly, drug delivery [35], and enzymatic reactions [36,37]. Unsaturated double bonds can be used as sites for cross-linking [38]. The aromatic ring structure can increase the hydrophobic properties. In addition, CA is also a valuable food preservative and a heat stabilizer in plastic. The presence of the –CH=CH–COOR chain in CA and its derivatives can facilitate the radical stabilization [39,40]. Thus, these modified polymers can be used as a versatile platform for surface engineering and modification [2,6,37,41].

This work reports the esterification of xylan and cinnamic anhydride in homogenous 1-allyl-3-methylimidazolium chloride (AMIMCl) ILs and their applications in the wet-end of papermaking, organic–inorganic composite films, and hydrogels.

## 2. Results and Discussion

### 2.1. Dissolution Mechanism of Xylan in Ionic Liquid

**Scheme 1:**
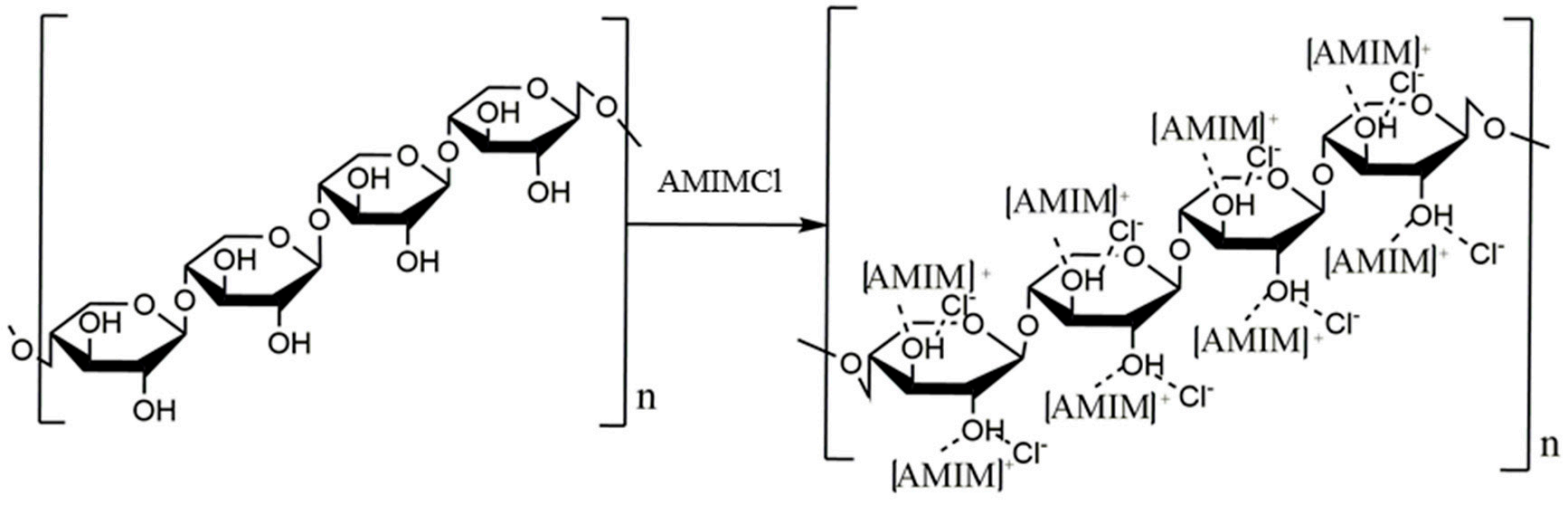
Proposed dissolution mechanism of xylan in 1-allyl-3-methylimidazolium chloride (AMIMCl).

The possible dissolution mechanism of xylan in AMIMCl ILs is shown in Scheme 1. The proposed dissolving mechanism confirms that both anions and cations are involved in the dissolution process [42]. As has been described by Zhang et al., the oxygen and hydrogen atoms of OH of xylan act as electron donors and electron acceptors to form electron donor–acceptor (EDA) complexes with ILs [43]. This interaction results in the separation of the hydroxyl groups of xylan, leading to the dissolution of xylan in ILs. The interaction between the hydroxyl group of polysaccharides and ILs is critical. After 60 min, xylan completely dissolves.

### 2.2. Effects of Reaction Conditions on the Degrees of Substitution (DS) of Cinnamic Anhydride Xylan (CAX)

Xylan modification was performed by esterification of hydroxyl groups on the backbone of xylan with CA using AMIMCl ILs as a homogeneous reaction medium in the presence of NaOH as catalyst.

Table 1 shows the extent of the chemical modification expressed by degrees of substitution (DS) when the reaction was carried out under various conditions. When the amount of alkali is greater than 0.025 M, an ideal catalytic effect is achieved beyond which excess alkali could not improve the reaction efficiency [44]. Through reactions 1–3 in Table 1, the catalytic efficiency of various catalysts can be observed. NaOH exhibits a stronger influence on the rate of acylation with CA. This is because the decomposition reaction of hemicelluloses by alkali is accelerated, and the addition of NaOH leads to an accretion of negative surface oxide ions, thus increasing esterification reactivity.

Reactions 4–8 in Table 1 show the effect of reaction time. The DS increased from 0.15 to 0.46 when the reaction time increased from 20 to 80 min; afterwards, the DS decreased significantly up to 100 min. This observation indicates that sufficient reaction times facilitate homogeneous esterification of xylan with CA, whereas excessive time may lead to the degradation of hemicelluloses and unwanted side reactions.

Reactions 9–13 explored the effect of reaction temperature. When the reaction temperature rose from 70 °C to 80 °C, the DS of the product dramatically increased from 0.15 to 0.45. A higher reaction temperature favors the compatibilization of reaction ingredients, swellability of xylan, diffusion of etherifying reagent, and mobility of the reactant molecules. However, when the reaction temperature was further raised to 110 °C, the DS of the esterified product decreased to 0.22. This is because the hemicellulose likely degraded.

Reactions 14–18 illustrate that the molar ratio of CA/xylan plays a vital role in the DS of CAX. A maximum DS of 0.57 was obtained with a molar ratio of CA/xylan that was 6:1. When the ratio increased to 8:1, the DS decreased to 0.37. Similar results showed that a higher concentration of esterification agent may result in cross-linking and other side reactions, thus reducing DS [18].

### 2.3. FT-IR Spectra Analyses

**Figure 1:**
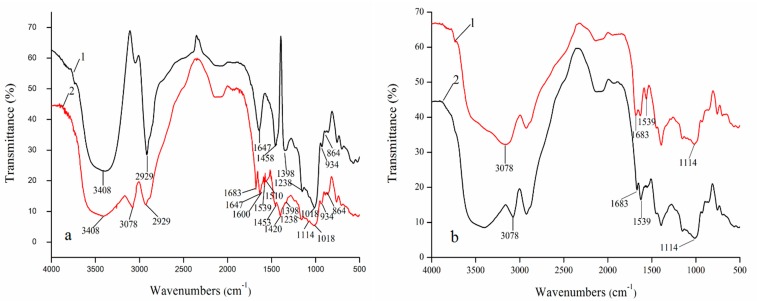
(**a**) FT-IR spectra of xylan (spectrum 1) and CAX (spectrum 2, sample 17 in Table 1); (**b**) FT-IR spectra of CAX with different DSs (spectrum 1, sample 17; and spectrum 2, sample 18).

The effect of chemical modification on the structure of xylan was interrogated by Fourier transform infrared spectroscopy (FT-IR) over 400–4000 cm^−1^. Figure 1a illustrates the FT-IR spectra of xylan (spectrum 1) and CAX (spectrum 2, sample 17 in Table 1) from AMIMCl ILs. The absorbance at 3408, 2929, 1647, 1458, 1398, 1238, 1116, 1018, 933, and 864 cm^−1^ is characteristic of xylan [21]. A sharp band at 864 cm^−1^ is assigned to β-glycosidic linkages between the sugar units [45]. The low intensity of the band at 933 cm^−1^ suggests the presence of arabinosyl units, attached only at position 3 of the xylopyranose constituents [33]. The absorption of 1463 and 1018 cm^−1^ belongs to the stretching vibration of C–H and C–O, respectively. A strong broadband at 3408 cm^−1^ is from the vibration band of O–H, while a symmetric C–H vibration band is at 2929 cm^−1^ [46]. Compared with xylan, three new absorbances at 1683 (C=O), 1539 (C=C), and 1114 cm^−1^ (–C–O–) appeared for CAX, indicating an esterification reaction. The significant decrease in the intensity of 3408 cm^−1^ (O–H) indicates that the acylation occurs between the hydroxyl groups of xylan and the carboxyl groups of the CA. The new absorbance peaks at 1600, 1510, and 1420 cm^−1^ indicate that benzene is grafted to CAX. Finally, the absence of peaks between 1850 and 1780 cm^−1^ demonstrates that the product does not contain unreacted CA.

Figure 1b illustrates the FT-IR spectra of CAX with different DSs. The intensity of the ester bands at 3078, 1683, 1539, and 1114 cm^−1^ increased with DS from 0.11 to 0.57. Accordingly, the intensity of the band at 3408 cm^−1^ for hydroxyl groups decreased with DS.

### 2.4. ^13^C-NMR Spectra

**Figure 2:**
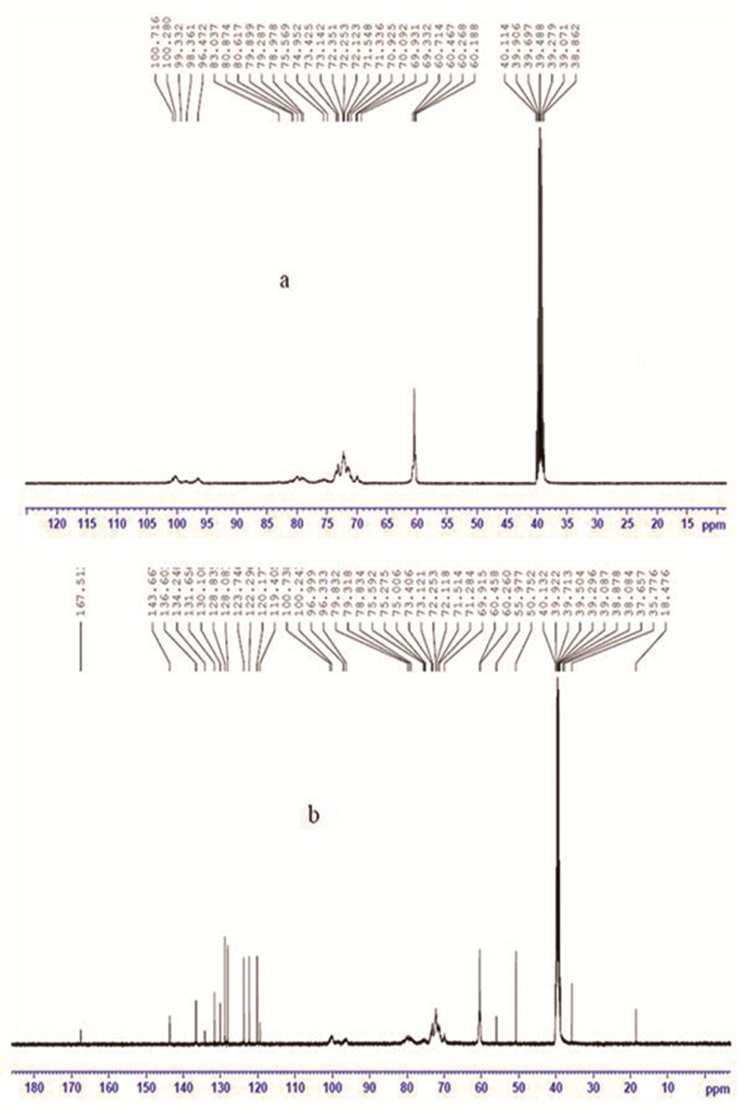
^13^C-NMR spectra of xylan (spectrum (**a**)) and CAX (spectrum (**b**), sample 17 in Table 1).

Figure 2 shows the ^13^C-NMR spectra of xylan (a) and CAX (b). In Figure 2, five strong signals at 100.3, 75.6, 72.3, 60.7, and 60.5 ppm are attributed to the C-1, C-4, C-3, C-2, and C-5 carbons of the (1→4)-linked β-d-Xylan units, respectively. In addition, the signals at 38.9 to 40.1 ppm are indicative of methylene carbon atoms.

Compared with the spectrum of xylan, new peaks emerged in the ^13^C-NMR of CAX. Signals between 119.4 and 143.7 ppm are attributed to carbon–carbon double bonds and the phenyl. The signals at 167.5 ppm represent ester bonds [47] in CAX, whereas the signals at 50.8, 56.0, and 60.3 ppm are oxygen atoms linked to methylene carbon atoms.

### 2.5. Thermal Analysis

**Figure 3:**
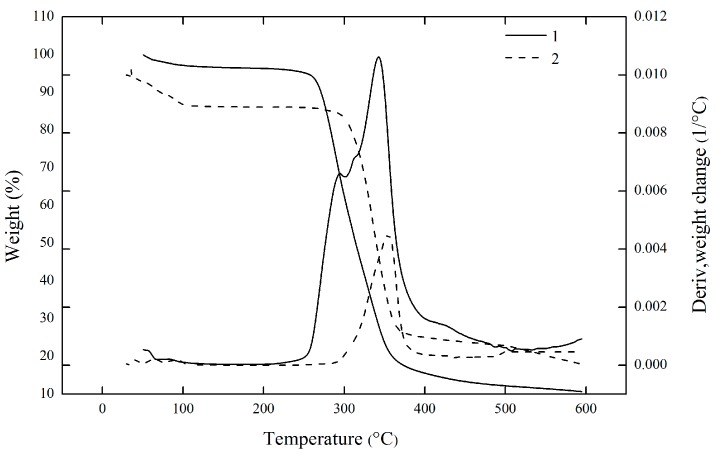
TGA/DTA curves of CAX (curve 1, sample 17 in Table 1) and xylan (curve 2).

Figure 3 shows typical TGA/DTA curves of xylan and CAX. The thermal stability of CAX was less than that of xylan. Rapid degradation of CAX and xylan started at 270 °C and 300 °C, respectively. The temperatures corresponding to the maximum weight loss rate of CAX and xylan were 356 °C and 365 °C, respectively. A weight loss of 50% occurred at 314 °C and 335 °C for CAX and xylan, respectively. A decreasing trend in the decomposition temperature indicated that the thermal stability of xylan decreased after esterification. This may be because more hydrogen bonds in xylan macromolecules were destroyed during chemical modification as well as the instability of the ester bond formed with cinnamic anhydride [48,49].

### 2.6. Applications of CAX

#### 2.6.1. Physical Properties of Paper

**Figure 4:**
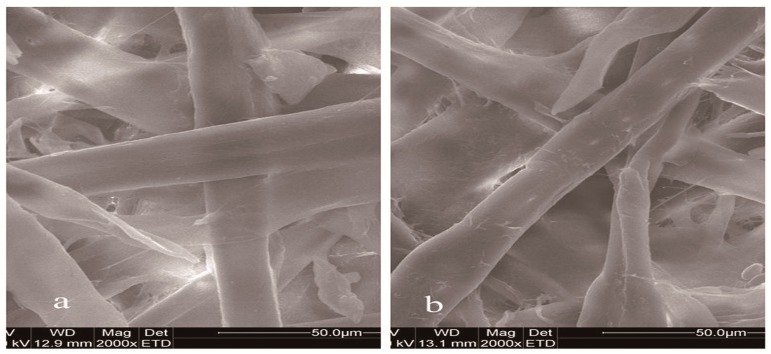
SEM of hand sheets of alkaline peroxide mechanical pulp (APMP) pulp/chemical pulp (1:1) without additives (**a**) and with the addition of CAX (**b**).

At a DS of 0.18, reaction time = 15 min, and alkaline peroxide mechanical pulp (APMP) pulp/chemical pulp = 1:1, desired sheet physical properties can be obtained. Increased sheet physical properties mainly require enhanced forces between fibers. Figure 4a,b show the fiber intertexture without and with additives. There are many interspaces among fibers within the control sample without additive (Figure 4a). However, packing between the fibers was very tight (Figure 4b), and because the polymers were on the surface, the fibers were very well bonded. In addition, the agent enhanced retention of fine fibers, and improved the uniformity of the surface of hand sheets.

#### 2.6.2. Organic–Inorganic Films

**Figure 5:**
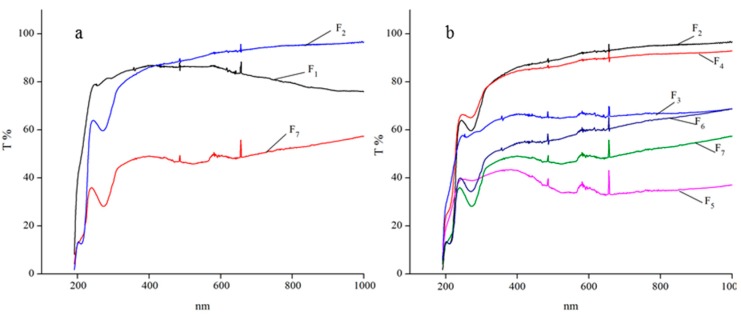
UV-vis spectra of composite films with different DS of XC and CAX. ((**a**): F_1_, F_2_; F_7_; (**b**): F_2_–F_7_; F_1_–F_7_ respectively representative of the DS: 0, 0.11, 0.18, 0.21, 0.33, 0.46, and 0.57).

Cinnamic acid and polyvinyl alcohol display an opportunity to occupy space in the food packaging materials. In Figure 5, seven films exhibit transmittance in the UV and visible ranges, a favorable effect for food protection. By contrast, a and b showed that a high DS was not favorable for transmittance. When the DS was 0.11, the UV and visible light transparency of the films was the highest. Yet, at a DS = 0.57, the transmittances were much lower. However, there was no discrimination in the behavior. The reason for this phenomenon is not known, but the results indicated that the transparencies of other samples are lower than that of F_2_ derivatives.

#### 2.6.3. Hydrogels and Heavy Metal Ion Adsorption

**Figure 6:**
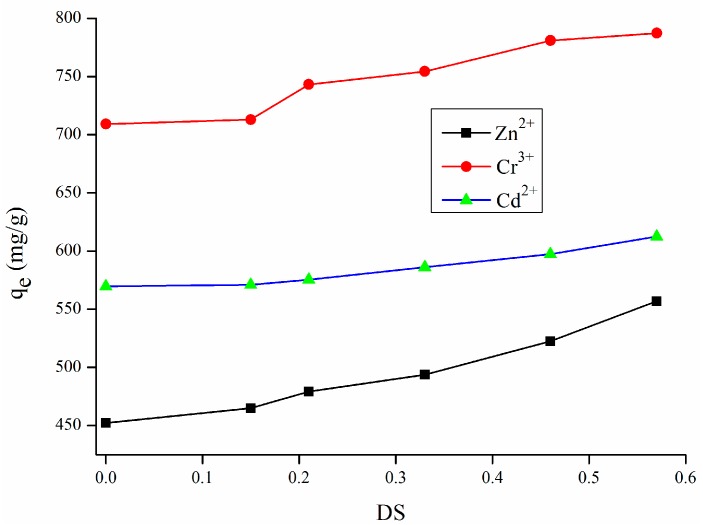
Effect of DS on the hydrogel adsorption for xylan-chain-g-acrylic acid (CAX-g-AA) hydrogel.

Hydrogels were prepared to test their adsorption of heavy metal ions (Cr^3+^, Cd^2+^, and Zn^2+^) at pH 5.5, 6.5, and 6.5. At an MBA/CAX ratio of 0.1 and AA/CAX ratio of 8, the hydrogel adsorption was shown to be best. The adsorption trends with DSs can be observed in Figure 6: not surprisingly, with an increase in DS, the adsorption capacity of different metal ions is significantly enhanced. For example, the adsorption capacity of Cr^3+^ increased from 709 mg/g at DS 0 to 787 mg/g at DS 0.57. The adsorption capacities of Cd^2+^ and Zn^2+^ also increased with DS, especially at 0.57.

## 3. Materials and Methods

### 3.1. Materials

Xylan was purchased from Chinese Medicine Group Chemical Reagent Co., Ltd. (Shanghai, China). Ethanol, LiOH, KOH, NaOH, Al_2_(SO_4_)_3_ and cinnamic anhydride were purchased from Sinopharm Chemical Reagent Factory, Shanghai, China. Alkaline peroxide mechanical pulp (APMP) was obtained from the Qilu University of Technology. 1-allyl-3-methylimidazolium chloride (AMIMCl) ionic liquid was purchased from Shanghai Cheng Jie Chemical Co., Ltd. (Shanghai, China).

### 3.2. Homogeneous Esterification of Xylan in AMIMCl

A typical procedure for the homogeneous esterification of xylan with CA in AMIMCl ILs is as follows. Dry xylan was added into ionic liquid (2.5%, *w*/*w*) at a ratio of 1:15 (*w*/*w*) in a three-necked flask with a magnetic stirrer under N_2_ at 80 °C for 80 min to completely dissolve xylan. The temperature was regulated and a specific amount of catalyst (KOH, NaOH, and LiOH, 0.025 g) and CA (the molar ratios of CA to xylan were 1:1, 2:1, 4:1, 6:1, 8:1) were added before reaction. The temperatures were 70, 80, 90, 100, and 110 °C, and the time varied from 20, 40, 60, 80, to 100 min. After reaction, the mixture was cooled to room temperature and precipitated with ethanol (95 wt %, 150 mL) under stirring for 60 min. Then, the mixture was centrifuged and the precipitate was washed with ethanol (95 wt %, 150 mL) twice. Finally, the precipitate was dried in a vacuum oven (45 °C) for 16 h. All experiments were done in duplicate with a standard error less than 4% [13,20,44].

### 3.3. Determination of Degree of Substitution

The degree of substitution (DS) of cinnamic anhydride xylan (CAX) was determined by acidimetric titration [18]. Two procedures determined the DS of CAX as follows: (a) Determination of Purity of CAX. CAX (0.25 g) was dissolved in 5 mL deionized water, then mixed with 1M HCl of 5 mL, and stirred until dissolved. An amount of 1 mL of phenolphthalein indicator was added into the mixture followed by dropwise addition of NaOH (1 M) with stirring until the red color disappeared. The mixture was poured into 95 wt % ethanol (50 mL) under stirring and then was settled for 15 min after centrifugation. The precipitate was washed with 80% ethanol four times, and washed one time with 95% ethanol (50 mL). Finally, the precipitate was dialyzed (cutoff = 3500 g mol^−1^) with distilled water and freeze-dried. (b) Determination of DS of CAX. The average values of DS of CAX were determined by acidimetric titration. CAX (0.05 g) was dissolved in 50 mL of distilled water with a magnetic stirrer. pH of the solution was adjusted to 8.00 with NaOH solution. H_2_SO_4_ (0.05 M) was used to adjust the solution pH to 3.74. The DS was based on the following equations:

(1) $$a=\;\frac{m^{\prime }}{m}$$

(2) $$B=\;\frac{2\mathrm{MV}}{am}$$

(3) $$\mathrm{DS}=\;\frac{0.132B}{1-0.1B}$$

where *a* is the purity of CAX. *m’* and *m* (g) are weights of CAX before and after purification, M = normality of H_2_SO_4_, V = mL of H_2_SO_4_ used to titrate sample, 0.132 (g mmol^−1^) is the molar mass of a xylose unit, 0.1 (g mmol^−1^) is the net increase in the mass of a xylose unit for each CAX substituted, *B* = mol g^−1^ of H_2_SO_4_ consumed per gram of product. All the titrations were carried out in triplicates and standard deviations were <4.0%.

### 3.4. Characterization of CAX

The solution-state ^13^C-NMR spectrum was carried out on a Bruker AVANCE III 400 MHz spectrometer (Bruker, Karlsruhe, Germany) operating in FT mode at 100.6 MHz. The sample (50 mg) was dissolved in 1 mL polyacrylonitrile-dimethyl sulfoxide solution. The ^13^C-NMR spectrum was recorded at 25 °C after 30,000 scans. A 30-pulse flipping angle, a 9.2 μm pulse width, a 1.36 s acquisition time, and 2 s relaxation delay time were used.

FT-IR transmission spectra of xylan and CAX were recorded on a Shimadzu Corporation IRPrestige-21 using a KBr pellet containing 1% sample over a range of 4000 to 500 cm^−1^. Thermal analysis was performed using thermogravimetric analysis (TGA) and differential thermal analysis (DTA) on a simultaneous thermal analyzer (Q-50, American TA Corporation, New Castle, PA, USA). The apparatus was continually flushed with N_2_. The sample was weighed between 9 and 11 mg and heated from room temperature to 600 °C at a heating rate of 5 °C/min.

### 3.5. Application of CAX

#### 3.5.1. Wet-End of Papermaking

The influence of CAX having different DS on the physical properties of hand sheets was tested. The following variables were chosen: aluminum sulfate as the absorption point, control of different DSs (0.11–0.57), homogeneization time (5–20 min), APMP pulp/chemical pulp (50–100%).

#### 3.5.2. Preparation of Organic–Inorganic Composite Films

Using CAX with different DSs, montmorillonite (MMT) and polyvinyl alcohol (PVA) were used to make the composite films. Scanning electron microscope (SEM), atomic force microscope (AFM), FT-IR, X-ray diffraction (XRD), and thermogravimetric analysis (TGA) were used to observe the morphology, structure, and thermal properties.

#### 3.5.3. Preparation of Hydrogel and Adsorption of Heavy Metal Ions

Xylan- and CAX-based hydrogels by graft co-polymerization of acrylic acid (AA) were prepared using 1-allyl-3-methylimidazolium (MBA) to study the adsorption of heavy metal ions (Cd^2+^, Cr^3+^, and Zn^2+^) from aqueous solutions. The effects of AA and MBA, DS, pH value, contacting time, and initial concentration of metal ion on the adsorption capacity were studied. The chemical structure, interaction between the hydrogel and metal ions, and the porous structure of cinnamon acidified xylan-chain-g-AA (CAX-g-AA) hydrogel were interrogated by FT-IR and SEM.

## 4. Conclusions

A new hemicellulose derivative, cinnamic anhydride esterified hemicellulose, was homogeneously synthesized in AMIMCl ionic liquid with several catalysts. The influence of the level of cinnamic anhydride, reaction temperature, time, and catalyst on the DS (0.11–0.57) of the products was investigated. The optimum reaction conditions were as follows: 0.025 g NaOH (catalyst/reactant), cinnamic anhydride/anhydroxylose molar ratio of 6:1, 80 °C, 80 min. FT-IR and ^13^C-NMR analysis confirmed the xylan derivatives. Thermal analysis showed that the thermal stability of the derivatives was reduced. Finally, application of the cinnamic anhydride esterification hemicellulose derivatives in the wet-end of papermaking, organic–inorganic composite films and hydrogel were shown. In wet-end papermaking, ideal paper performance can be obtained when the DS is 0.18, whereas when the DS was 0.11 and 0.57, adsorption of the composite films and hydrogel was best, respectively. These new CA-based hemicellulosic materials therefore offer new opportunities for sorbency, hosting/delivery, wound repair, food packaging, and cosmetics among others.

## Figures and Tables

**Table 1:** The degrees of substitution (DS) of the cinnamic anhydride xylan (CAX) obtained by the reaction of xylan with cinnamic anhydride (CA) in various reaction conditions.

Samples ^a^	Conditions				Esterification of Hemicellulose
	Esterification Molar Ratio of CA/Xylan ^b^	Reaction Time (min)	Temperature (°C)	Catalyst (0.025 mol/L)	DS ^c^
1	2:1	80	80	NaOH	0.15
2	2:1	80	80	KOH	0.11
3	2:1	80	80	LiOH	0.06
4	2:1	20	80	NaOH	0.15
5	2:1	40	80	NaOH	0.18
6	2:1	60	80	NaOH	0.21
7	2:1	80	80	NaOH	0.46
8	2:1	100	80	NaOH	0.25
9	2:1	80	70	NaOH	0.15
10	2:1	80	80	NaOH	0.45
11	2:1	80	90	NaOH	0.36
12	2:1	80	100	NaOH	0.29
13	2:1	80	110	NaOH	0.22
14	1:1	80	80	NaOH	0.15
15	2:1	80	80	NaOH	0.13
16	4:1	80	80	NaOH	0.33
17	6:1	80	80	NaOH	0.57
18	8:1	80	80	NaOH	0.37

^a^ All samples were prepared at 1.25% concentration of xylan in ionic liquid (based on weight); ^b^ Molar ratio of cinnamic anhydride/xylose unit in xylan, xylose unit M = 150; ^c^ Based on the assumption that all of the xylasns are converted to di-acylated xylan (DS, 2.00). If no reaction occurred, and all of the xylan were recovered unreacted, the DS value would be 0.00.
